# Supplementary material for: The response of soil Olsen-P to the P budgets of three typical cropland soil types under long-term fertilization
Source: PLoS One. 2020 Mar 18;15(3):e0230178. doi: 10.1371/journal.pone.0230178 (PMC7080265; doi:10.1371/journal.pone.0230178)
Supplement: Attachment — Submitted filename: Response to Reviewers.docx [file pone.0230178.s002.docx]

Dear reviewers,

Thank you very much for your comments of our manuscript entitled “The response of soil Olsen-P to the P budgets of three typical cropland soil types under long-term fertilization”. Those comments are very valuable for revising and improving our paper. Based on your suggestion, we made correction carefully. The responds to the comments are as follows:

1. About the English grammar and writing, I have the manuscript polished by AJE and a teacher from England in CAAS help me. The manuscript is revised submission with new line and page numbers in the text, some grammar and spelling errors had also been corrected.
2. Some parts are unnecessary in the manuscript have been deleted, such as figures, funding statement.
3. **Comment: “Several parts of the manuscript are very similar with the papers…….In order to reduce the degree of overlap, I suggest you carefully rewrite the text in these sections to ensure that it is original.”**

**Answer:** I have rewrite my text especially the part of “**Materials and methods**” by my own words to reduce the degree of overlap. Some descriptions are close to the paper of Qihua Wu, but I have tried to not the same as hers.

4. **Figure 1 (partially) and Tables 1 and 2 have already published and therefore the authors must use the appropriate citations.**

**Answer:** The location of the three long-term experiment sites in the manuscript is the same with paper “Characterizing differences in the phosphorus activation coefficient of three typical cropland soils and the influencing factors under long term fertilization”. And the three palaces I have used citations.

5. **“Please clarify what is the contribution of the residual phosphorus on the soil phosphorus budget for the three soil types?”**

**Answer:** When the amount of P fertilizers was higher the crop uptake, soil P showed a surplus. Soil P surplus was increasing by an annual growth rate of 11%. Withholding P applications, it may be feasible that accumulated soil P will built up soil P reserves for crop growth [28]. Thus, taking advantaging of soil accumulated P not only can reuse and save P resource, but also can reduce the risk of environmental pollution.

6. **“A great deal of fixed P in Qiyang dissolved at the lower pH, and it ran off easily with more precipitation’’. How? Please explained the mechanism of P release.”**
   **Answer:** I think this sentence was wrong to write about pH，so I change it into “The result might be related to the amount of precipitation for Al-P, Fe-P and O-P at the soil surface was easily lost with rainfall (surface runoff) in Hunan province, especially for upland red soil The precipitation in Eutric Cambisol was two times that of Calcaric Cambisol, so soil P loss with rainfall in Eutric Cambisol could be higher than that in Calcaric Cambisol.”
7. **“Soil containing large quantities of clay fix more P than soil with low clay contents [42,43]. Thus, a high clay content might be another reason for the significant increase in soil Olsen-P with the low P surplus under NPK and NPKS in Gongzhuling’’**. This is a contradiction. Fixation of P by Fe and Al oxides in clay soils is a factor that restrains P availability and the efficiency of P fertilizers. Please re-write the sentence.
   **Answer:** Yes, you are right. I have write the sentences as “In alkaline calcareous soils with high pH values, the inorganic phosphates (primarily as HPO_4_^2-^) in the soil solutions combine with Ca to form a series of Ca-P compounds [40,41]. In Calcaric Cambisol of our study, Ca_10_-P and O-P which were hard to decompose might be the main compounds of soil P. Soil containing large quantities of clay fix more P than soil with low clay contents [42, 43], the soil clay content was strongly related to increasing the Olsen-P across seven long-term experimental sites in China, except for the Huan site [4]. But in Calcaric Cambisol, the content of clay was low, so the adsorption of P might be low, precipitation could be a main way for P fixing in the soil of Calcaric Cambisol.
8. **‘’In acidic soils with low pH values, inorganic phosphates (primarily as H2PO4-) combine with Fe and Al to form different Fe-P and Al-P compounds [37], lowering the availability of the soil P’’. Add also the more recent publication:
   https://doi.org/10.1016/j.catena.2019.104106
    ‘’…..and applying gypsum was recommended to improve the soil pH’’ !!! . Gypsum does not change soil pH since is a neutral salt and not a liming agent.**

**Answer:** I have delated the sentence of **‘’In acidic soils with low pH values, inorganic phosphates (primarily as H2PO4-) combine with Fe and Al to form different Fe-P and Al-P compounds [37], lowering the availability of the soil P’’,** for I did not discuss a lot about this aspect.

Yes, you are right. Gypsum does not change soil pH, actually, they applied lime in the experiment site to increase soil pH.

9. **Ferralic Cambisol (as in the text) or Eutric Cambisol (as in the table)??
   Answer:** It is Eutric Cambisol, and I have corrected it in the text.
10. **As written the paper is only of local interest; therefore, I encourage the authors to replace the locations with soil type according to FAO classification through the manuscript
    For example, replace the order Gongzhuling > Qiyang > Zhengzhou with Luvic Phaeozems > Ferralic Cambisol > Calcaric Cambisol**

**Answer:** Thank you for your good comments, I have change the name of soil by FAO classification instead of the name of sites.

11. **Conclusion is very weak. As currently written is just a repetition of the results section. The authors should rewrite this section emphasizing the importance of their findings on the soil phosphorus management in relation to soil type and P budget.**
    **Answer:** I have rewritten the conclusion part according to your advice.

Once again, many thanks for your generous help with our manuscript.

Your’s sincerely,

Shuxiang Zhang
